# Comparative genomics unveils extensive genomic variation between populations of *Listeria* species in natural and food-associated environments

**DOI:** 10.1038/s43705-023-00293-x

**Published:** 2023-08-19

**Authors:** Jingqiu Liao, Xiaodong Guo, Shaoting Li, Sai Manohar Balu Anupoju, Rachel A. Cheng, Daniel L. Weller, Genevieve Sullivan, Hailong Zhang, Xiangyu Deng, Martin Wiedmann

**Affiliations:** 1 Department of Food Science, Cornell University, Ithaca, NY USA; 2 Department of Civil and Environmental Engineering, Virginia Tech, Blacksburg, VA USA; 3 School of Biomedical and Pharmaceutical Sciences, Guangdong University of Technology, Guangzhou, Guangdong China; 4 Department of Computer Science, Virginia Tech, Blacksburg, VA USA; 5 Department of Food Science and Technology, Virginia Tech, Blacksburg, VA USA; 6 Department of Biostatistics and Computational Biology, University of Rochester Medical Center, Rochester, NY USA; 7 Department of Business Information Technology, Virginia Tech, Blacksburg, VA USA; 8 Center for Food Safety, University of Georgia, Griffin, GA USA

**Keywords:** Population genetics, Environmental microbiology

## Abstract

Comprehending bacterial genomic variation linked to distinct environments can yield novel insights into mechanisms underlying differential adaptation and transmission of microbes across environments. Gaining such insights is particularly crucial for pathogens as it benefits public health surveillance. However, the understanding of bacterial genomic variation is limited by a scarcity of investigations in genomic variation coupled with different ecological contexts. To address this limitation, we focused on *Listeria*, an important bacterial genus for food safety that includes the human pathogen *L. monocytogenes*, and analyzed a large-scale genomic dataset collected by us from natural and food-associated environments across the United States. Through comparative genomics analyses on 449 isolates from the soil and 390 isolates from agricultural water and produce processing facilities representing *L. monocytogenes*, *L. seeligeri*, *L. innocua*, and *L. welshimeri*, we find that the genomic profiles strongly differ by environments within each species. This is supported by the environment-associated subclades and differential presence of plasmids, stress islands, and accessory genes involved in cell envelope biogenesis and carbohydrate transport and metabolism. Core genomes of *Listeria* species are also strongly associated with environments and can accurately predict isolation sources at the lineage level in *L. monocytogenes* using machine learning. We find that the large environment-associated genomic variation in *Listeria* appears to be jointly driven by soil property, climate, land use, and accompanying bacterial species, chiefly representing *Actinobacteria* and *Proteobacteria*. Collectively, our data suggest that populations of *Listeria* species have genetically adapted to different environments, which may limit their transmission from natural to food-associated environments.

## Introduction

Bacterial genomes, including both core genomes (genes present in all individuals) and accessory genomes (genes not shared by all individuals), can be highly versatile within species due to gene gain and loss and homologous recombination mediated by environmental selection and dispersal [1–4]. Such genomic variation enables bacterial species (mostly non-pathogenic bacteria) to live in a wide array of ecological dimensions, including environmental conditions with different sources of carbon and inorganic nutrients [5]. While some human pathogens (e.g., *Bacillus anthracis*, *Clostridium* spp., *Listeria monocytogenes*, *Yersinia pestis, Burkholderia pseudomallei*, and *Francisella tularensis*) can also survive in natural environments [6], our understanding of their genomic variation across different environments is limited due to a lack of intensive investigations in natural environments compared to human-associated environments [7–9]. This is a missed chance to enhance an understanding of the ecological mechanisms underlying the adaptation of pathogens to non-human environments and to better inform public health surveillance for infectious diseases, such as inferring the likelihood of strains transmitting from natural environments to human-associated environments.

*Listeria*, a Gram-positive, facultatively anaerobic, non-spore-forming bacterial genus vital to food safety, serves as an opportunity to study genomic variation between natural and human-associated environments in bacteria important to public health. The members of *Listeria* are widely distributed in natural environments as well as agricultural soil, water, and food processing facilities [10–12]. Two *Listeria* species—*L. monocytogenes* and *L. ivanovii*—are considered facultative pathogens. While other species are non-pathogenic [13], these species (e.g., *L. seeligeri*, *L. innocua*, and *L. welshimeri*) are often tested in the food industry because it is considered evidence of conditions that may facilitate *L. monocytogenes* contamination [14, 15]. Studying the genomic variation of *Listeria* species can thus provide insights into its transmission from natural environments to food-associated environments and foods, which is particularly important for food items such as fresh produce, where no-kill steps are used to inactive pathogens that would be introduced at any point of the food chain.

Food-associated environments normally have a wide range of stressors present, including low pH, low water activity, low temperature, high salinity, sanitizers (e.g., quaternary ammonium), and antimicrobial compounds (e.g., nisin) [16]. *Listeria* species, including *L. monocytogenes*, have developed diverse stress response mechanisms, including a σ^B^-dependent general stress response regulon and two stress survival islets (SSI-1 and SSI-2), which facilitate growth at low pH and high salt concentrations (SSI-1) as well as growth at high pH and in oxidative conditions (SSI-2) [17, 18]. In nature, *Listeria* lives a saprotrophic lifestyle, playing a vital role in decomposition and nutrient cycling in an ecosystem [19]. Compared to food-associated environments, natural environments provide bacteria with relatively favorable and stable conditions with moderate alterations in physicochemical properties [20, 21]. However, how *Listeria* species differentially adapt to and transmit across these two types of environments is largely unknown.

Here, we leveraged genomic data of 449 *Listeria* isolates we obtained from a recent large-scale soil collection across the United States [22] and further sequenced genomes of 390 *Listeria* isolates obtained from different food-associated environments, including (i) agricultural water [23, 24] and (ii) produce processing facilities [25, 26]. These isolates represent the foodborne pathogen *L. monocytogenes* and three common non-pathogenic species (*L. seeligeri*, *L. innocua*, and *L. welshimeri*). Through in-depth comparative genomics analyses, we identified strong associations in phylogeny, core and accessory genomes, plasmids, and stress survival islets with natural or food-associated environments. We also developed machine learning models that can accurately predict isolation sources for *L. monocytogenes* lineages using core genome data and identified potential selective abiotic and biotic pressures driving the genomic association with the natural environment. Our findings suggest that populations of *Listeria* species have distinct niches in natural and food-associated environments, potentially limiting the likelihood that *Listeria* strains frequently transmit between these two environments.

## Materials and methods

### *Listeria* isolates from natural and food-associated environments

The 839 *Listeria* isolates used in this study (Table S1) included (i) 449 isolates collected from soil in the natural environment across the United States [22], (ii) 115 previously reported isolates from agricultural water [23, 24], and (iii) 275 isolates from produce processing facilities [27]; the term “food-associated environment” is used to refer to the source of isolates in categories (ii) agricultural water and (iii) produce processing facilities. The 449 isolates representing set (i) included 177 *L. monocytogenes* isolates representing lineages I, II, and III (*n* = 12, 39, and 126, respectively), *L. seeligeri* (*n* = 98), *L. innocua* (*n* = 33), and *L. welshimeri* (*n* = 141) isolates collected from soil in the natural environment across the US in 2018. While the original set reported by Liao et al., [22] also included isolates representing other species, these isolates were not included in the study reported here as none of these species were represented in the food-associated isolate set. The 115 isolates from set (ii) included 54 *L. seeligeri*, 19 *L. innocua*, and 42 *L. welshimeri* isolates collected from agricultural water in NY and AZ between 2017 and 2018. The 275 isolates from set (iii) included 176 *L. monocytogenes* isolates representing lineages I, II, and III (75, 68, and 33 isolates, respectively) as well as 47 *L. seeligeri*, 37 *L. innocua*, and 15 *L. welshimeri* isolates; these isolates were collected from produce processing facilities (packing houses and fresh-cut facilities) in the United States between 2017 and 2018.

### Environmental data and 16 S rRNA gene amplicon sequencing

Previously reported environmental variables [22], including latitude, longitude, soil property (17 variables), climate (4 variables), and surrounding land use (10 variables), were included in the ecological analyses for *Listeria* from the soil. Soil samples positive for *Listeria* (311 samples in total; Table S2) and an identical number of negative samples (311), which had previously been used for soil property measurements [22], were used for 16 S rRNA gene sequencing in this study. Soil for each sample was sieved using a sterile 2 mm sieve. Total DNA was extracted from each sample using QIAGEN Power Soil Pro kits (12888-100). DNA was stored at −80 °C until further processing. The V4 region of the 16 S rRNA gene was amplified according to methods detailed in the Supplementary Information. Sequencing was performed on a MiSeq using a 2 × 250 bp paired-end read run. The number of raw reads among all 622 samples ranged from 8599 to 59,425 (Table S2). Raw reads were processed using QIIME2 following the online tutorials (https://docs.qiime2.org/2020.2/tutorials/). Methods for processing raw reads (e.g., drawing alpha rarefaction curve, Fig. S1) and identification of operational taxonomic units (OTUs) are detailed in the Supplementary Information.

### Whole genome sequencing, phylogeny, cgMLST, and genome annotation

Whole genome sequence data for *Listeria* isolates in sets (i) and (iii) have previously been reported ([22, 27], respectively). Methods for DNA extraction, whole genome sequencing, genome assembling, and quality control of isolates detailed previously in Liao et al., [22] were used here to sequence isolates from set (ii). Core SNPs for genomes of each species were determined using kSNP3 3.1.2 [28]. Phylogenetic trees of each species were constructed using RAxML-8.2.12 [29] with 500 bootstrap repetitions based on the core SNPs. A GTR + G (General Time Reversible + gamma distribution) substitution model determined by jModelTest [30] with ascertainment bias correction was used in the tree construction. Core genome multilocus sequence typing (cgMLST) analysis was conducted using an in-house pipeline along with the cgMLST database available at the BIGSdb-Lm platform [31] (https://bigsdb.pasteur.fr/listeria) to assign allelic types for each *L. monocytogenes* genome. Genome annotation and identification of accessory genes were performed using methods detailed in Liao et al., [22].

### Detection of plasmid, stress survival islets, and virulence genes

Plasmids were detected by PlasmidFinder 2 using an identity cutoff of 0.6 [32]. Predicted plasmids were grouped into plasmid families (e.g., Inc18) and plasmid groups (e.g., rep13). Plasmid sequences were extracted from genomes for each plasmid group and alisgned using muscle 5.1. For each of the plasmid groups that harbored by more than three genomes (i.e., rep25, rep26, repUS25, and repUS43), a gene tree was constructed using RAxML-8.2.12 [29] with 1000 bootstrap repetitions based on aligned plasmid sequences. A GTR + G substitution model determined by jModelTest [30] was used in the tree construction. Genomes for all *Listeria* species were used to detect the presence of SSI-1, a five‐gene islet (lmo0444 - lmo0448), and SSI-2, a two-gene islet (lin0464 - lin0465). Genomes for all *L. monocytogenes* genomes were further used to determine the presence of selected virulence genes, including genes found in *Listeria* pathogenicity island 1 (LIPI-1; *prfA*, *plcA*, *hly*, *mpl*, *actA*, *plcB*), *Listeria* pathogenicity island 3 (LIPI-3; *llsAGHXBYDP*), *Listeria* pathogenicity island 4 (LIPI-4; *LM9005581_70009* - *LM9005581_70014*) as well as genes encoding selected different internalins (*inlAB,C,E,F,G,H,J,K,I,P*). SSI genes and these selected virulence genes were detected using BLASTN with default settings against reference databases available at the BIGSdb-Lm platform [31]. A premature stop codon was considered present in a given gene if a stop codon was detected before the last codon of the gene within the minimal length of the reference sequences. Genes were defined as (i) putative functional when coverage was >0.8 and no premature stop codon was present, (ii) putative non-functional when 0.3 ≤ coverage <0.8 or premature stop codon was present, and iii) absent when no hits were observed in BLASTN or coverage was <0.3. A coverage of 0.3 and 0.8 was chosen as the cutoffs because (i) the multi-domain structure of proteins is most likely preserved when using a coverage of 0.8 [33], and (ii) at least 0.3 or less query coverage has been recommended to identify genes that span contigs and/or touch gaps [34]. When calculating the presence/prevalence of a given gene across genomes, only putative functional genes are included in the calculation.

### Identification of source-associated genes and enriched COG functions

Within each species, associations between isolation sources and the presence of (i) virulence genes (*L. monocytogenes* only), (ii) plasmids, (iii) stress survival islets, and (iv) accessory genes were identified by Fisher’s exact tests, using rpy2 in Python 3.6.8. A Benjamin and Hochberg (BH) false discovery rate (FDR) correction for multiple testing was applied; items (e.g., accessory genes) with an FDR-adjusted *P* < 0.05 were determined to be source-associated. The correlation between source-associated orthologous genes and plasmid family Inc18 was assessed by Phi correlation analysis, using sklearn.metrics in Python 3.6.8. A binomial distribution model detailed in the Supplementary Information was used to identify clusters of orthologous gene (COG) function categories that were significantly enriched among source-associated accessory genes for each species and each *L. monocytogenes* lineage.

### Machine learning models to predict isolation source using cgMLST data

The gradient boosting algorithm LightGBM implemented in scikit-learn in Python 3 was used to develop predictive models for isolation sources of *L. monocytogenes* lineage I, II, and III using the presence/absence of cgMLST allelic profile of each lineage as features. Alleles that were present or absent in more than 99% of the samples were considered redundant and were excluded from the feature matrix. In LightGBM, the learning rate parameter was set to 0.05, the max_depth parameter was set to 10, and the num_leaves parameter was set to 80. All the other hyper-parameters are set to default values. All machine learning models were tested by twofold cross-validation. This method divides the dataset into two sections, each having a similar proportion of samples from each class as the entire set. This guarantees that the training and testing sets within each fold mirror the uneven distribution of the original dataset. In each fold, one section is used for training and the other for testing. The average value calculated across the two folds is used to assess the performance of the model. The area under the curve of the receiver operating characteristic (auROC) and the area under the curve of precision-recall (auPR) were calculated and visualized to measure the accuracy of the modes. The LightGBM feature importance score was computed using the internal LightGBM function “feature_importances_ .”This was computed for each fold within the 2-fold cross-validation, resulting in an importance score per feature from each fold. The average of the feature importance scores from the two folds was calculated to generate an overall importance score for each feature in each model.

### Identification of important abiotic variables

A partial Mantel test was performed between each environmental variable and average nucleotide identity (ANI) of genomes for each *Listeria* species and each *L. monocytogenes* lineage, using vegan in R 3.6.0 (999 permutations) by controlling geographical variables. ANI and geographic distance were calculated using methods reported by Liao et al., [22]. Environmental dissimilarity was calculated in Euclidean distance. A BH FDR correction was applied for correcting multiple partial Mantel tests. Variables with an FDR-adjusted *P* < 0.05 were defined as significant ecological variables and were included to assess the importance in predicting the ANI of *Listeria* in a random forest model using randomForest in R 3.6.0 following the procedures detailed in Liao et al., [22].

### Bacterial diversity and co-occurrence network

OTU richness and the Shannon-Wiener diversity index were calculated using skbio in Python 3.6.8. *t*-tests were performed to compare OTU richness and Shannon-Wiener diversity between samples positive and negative for *Listeria*. The co-occurrence of bacterial species and each *Listeria* species and each *L. monocytogenes* lineage was initially assessed by Phi correlation analysis using sklearn.metrics in Python 3. Bacterial species with a Phi correlation coefficient (r) >0.2 or <−0.2 with each *Listeria* taxon were defined as bacterial taxa that tend to have similar and dissimilar habitat preferences, respectively; these species were included in the co-occurrence network analysis. Networks of co-occurring bacterial species for each *Listeria* taxon were constructed using ggraph in R 3.6.0.

## Results

### Core genomes of *Listeria* species strongly differ between natural and food-associated environments and can accurately predict isolation sources at a lineage level

**Fig. 1:**
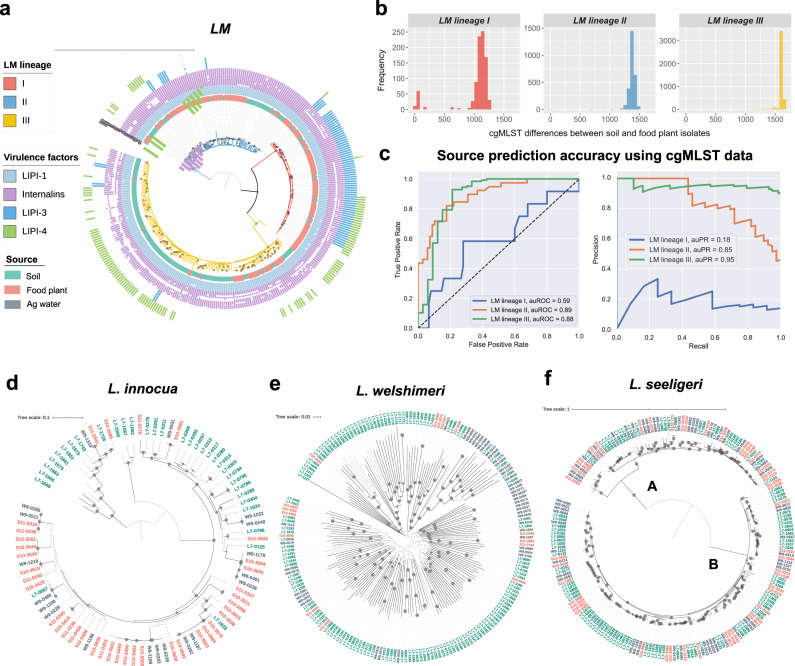
Phylogenies and core genomes of *Listeria* species are associated with isolation sources. **a** Maximum likelihood phylogenetic tree for *L. monocytogenes* based on core SNPs of 177 isolates from soil (tips in green) and 176 isolates from produce processing facilities (food plant) (tips in red) with 500 bootstrap repetitions. Bootstrap values >70% are indicated by gray circles on the bifurcation nodes. The tree was rooted by the midpoint. Branches are color-coded by *L. monocytogenes* lineages. The tree is annotated by the presence/absence of virulence genes. The presence/absence gene matrices from the inner to the outer represent (i) genes located in the pathogenicity islands LIPI-1 (*prfA*, *plcA*, *hly*, *mpl*, *actA*, *plcB*), (ii) genes coding for internalins (*inlABCEFGHJKIP*), and (iii) genes located in the pathogenicity islands LIPI-3 (*llsAGHXBYDP*) and LIPI-4 (*LM9005581_70009* to *LM9005581_70014*). A filled box represents the presence of a putative functional gene; an empty box represents a non-functional gene (i.e., being truncated or having premature stop codons); and a white box represents the absence of the gene. **b** Histograms showing the distribution of cgMLST allelic mismatches between isolates from soil and produce processing facilities (food plant) for *L. monocytogenes* (LM) lineage I (red), II (blue), and III (yellow). **c** ROC and PR curves for binary classifiers trained on cgMLST allelic profiles of LM lineage I, II, and III isolates. auROC: area under the curve of the receiver operating characteristic, auPR: area under the curve of precision-recall. Maximum likelihood phylogenetic tree of (**d**) *L. innocua*, (**e**) *L. welshimeri*, and (**f**) *L. seeligeri* based on the core SNPs of isolates of each species; isolates were obtained from soil, agricultural (ag.) water, and produce processing facilities (“food plant”). Trees were constructed based on 1000 bootstrap repetitions and were rooted by midpoint. Labels of isolates are color-coded by sources. Bootstrap values >70% are indicated by gray circles on the bifurcation nodes.

To assess the relationship between the core genome of *L. monocytogenes* (which entails genomes of all three *L. monocytogenes* lineages identified in this study - lineage I, II, and III) and isolation sources (soil versus food-associated), we first constructed a core-SNPs-based phylogenetic tree of 177 *L. monocytogenes* isolates from soil and 176 *L. monocytogenes* isolates from produce processing facilities (no *L. monocytogenes* isolates were available from agricultural water) (Fig. 1a). Results showed that *L. monocytogenes* lineages I and II were significantly predominated by isolates from produce processing facilities, while lineage III was predominated by soil isolates (Fisher’s exact *P* < 0.001). In addition, both lineages II and III showed environment-associated subclades (i.e., subclades composed of isolates mainly from one environment) (*P* < 0.01). Consistent with these findings, cgMLST identified a small number of isolate pairs (one isolate from soil and one from produce facilities) in *L. monocytogenes* lineages that were closely related (<50 allelic mismatches), including 42, 5, and 1 pair(s) within lineages I, II, and III, respectively (Fig. 1b, Table S3). These pairs included 31, 5, and 1 isolate(s) from produce facilities that were closely related to one or more soil isolates for lineages I, II, and III, respectively. These results show that the core genome of *L. monocytogenes* is strongly associated with the types of environments and may have the power to predict isolation sources at a lineage level.

To test the hypothesis regarding the predictability of the isolation sources of *L. monocytogenes* lineages using core genome data, we employed a machine learning algorithm, gradient boosting, to classify isolation sources for each lineage using cgMLST profiles as features. Our model obtained superior accuracy for lineage II and III (auROC = 0.89, auPR = 0.85 and auROC = 0.88, auPR = 0.95, respectively) and limited accuracy (auROC = 0.59, auPR = 0.18) for lineage I (Fig. 1c). Among the top 50 most important features (core genes) for predicting the isolation sources of each lineage (Fig. S2), a number of genes encode cell surface proteins, including PTS mannose transporter subunit IIC, PTS glucose transporter subunit IIA, PTS fructose transporter subunit IIA, and flagellar basal body rod protein FlgC for lineage I (Table S4), flagellar basal-body rod protein FlgB, PTS mannitol transporter subunit IIA, sugar ABC transporter permease, formate transporter, PTS sugar transporter subunit IIB, and PTS beta-glucoside transporter subunit IIB for lineage II (Table S5), and PTS sugar transporter subunit IIA, PTS beta-glucoside transporter subunit IIABC, and flagellar basal body rod modification protein for lineage III (Table S6). These results suggest that, enabled by machine learning techniques, core genome data are highly predictive for isolation sources for *L. monocytogenes* at a lineage level, and core genes involved in cell surface functions are strongly associated with isolation sources.

To further understand the relationship between core genomes and isolation sources in other *Listeria* species, we assessed the genetic relatedness among *L. seeligeri*, *L. innocua*, and *L. welshimeri* isolates from the soil, agricultural water, and produce processing facilities based on core SNPs; the numbers of isolates from the three environments were 98, 54, and 47 for *L. seeligeri*, 33, 19, and 37 for *L. innocua*, and 141, 42, and 15 for *L. welshimeri*, respectively. Based on the phylogenetic trees, isolates of *L. innocua* and *L. welshimeri* (which represented 4 and 5 major clades, respectively) strongly clustered by environments (Fig. 1d, e; Fisher’s exact *P* < 0.001 for both species). *L. seeligeri* had two major subclades (A and B) with a mixture of isolates from the soil, agricultural water, and produce processing facilities, and isolates from soil were significantly over-represented in subclade B compared to subclade A (Fig. 1f; *P* < 0.01). Consistent with these results, we detected a small number of closely related isolate pairs (a core SNP difference <50) between natural and food-associated environments (i.e., soil vs agricultural water, or soil vs produce processing facilities) in *L. seeligeri*, *L. innocua* and *L. welshimeri* (22, 9 and 0 pairs, respectively; Fig. S3, Table S7). SNP differences rather than cgMLST differences were used for these species because the cgMLST scheme is only available for *L. monocytogenes*. These results show that *L. seeligeri*, *L. innocua*, and *L. welshimeri* isolates from natural and food-associated environments are not closely related.

### Accessory genomes of *Listeria* species, particularly genes involved in cell envelope synthesis and carbohydrate metabolism, are significantly associated with isolation sources

**Fig. 2:**
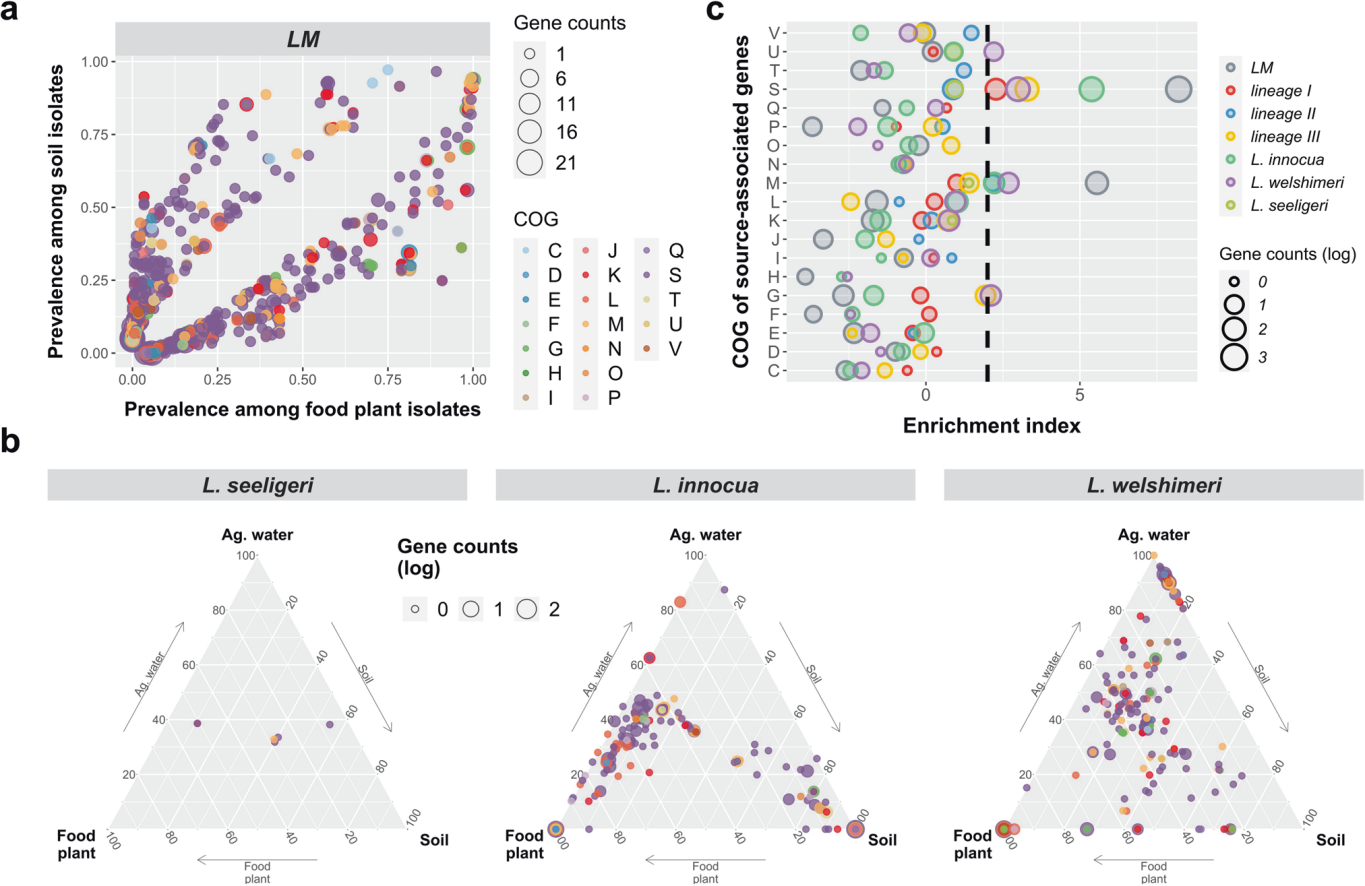
Source-associated accessory genes and enriched COG functions in *Listeria* species. Prevalence of source-associated accessory genes (**a**) between soil and produce processing facility (food plant) isolates of *L. monocytogenes* (*LM*) and (**b**) among soil, agricultural (ag.) water, and food plant isolates of *L. seeligeri*, *L. innocua*, and *L. welshimeri*. Dots are color-coded by COG functional categories. The size of the dots is in proportion to the logarithm 10 of the number of genes annotated as one COG category. Abbreviations of COG categories are described below. C: Energy production and conversion; D: Cell cycle control, cell division, chromosome partitioning; E: Amino acid transport and metabolism; F: Nucleotide transport and metabolism; G: Carbohydrate transport and metabolism; H: Coenzyme transport and metabolism; I: Lipid transport and metabolism; J: Translation, ribosomal structure, and biogenesis; K: Transcription; L: Replication, recombination, and repair; M: Cell wall/membrane/envelope biogenesis; N: Cell Motility; O: Posttranslational modification, protein turnover, chaperones; P: Inorganic ion transport and metabolism; Q: Secondary metabolites biosynthesis, transport, and catabolism; S: Function unknown; T: Signal transduction mechanisms; U: Intracellular trafficking, secretion, and vesicular transport; V: Defense mechanisms. **c** Enrichment analysis of COG functional categories for source-associated genes in *LM* (gray), *LM* lineage I (red), II (blue), and III (yellow), *L. seeligeri* (brown), *L. innocua* (green), and *L. welshimeri* (purple). Enrichment index >2 (black dashed line) indicates that the COG category is significantly enriched (*P* < 0.05). The size of the circle is in proportion to the logarithm of the number of genes annotated as one COG category.

Given that the core genome of each *Listeria* species is associated with isolation sources, we hypothesized that the accessory genome differs by the environment as well. To test our hypothesis, we employed Fisher’s exact tests to identify environment-associated accessory genes of *Listeria* species based on the presence/absence of a given gene in natural and food-associated environments. We identified 902 accessory genes across all *L. monocytogenes*, as well as 50, 36, and 195 accessory genes in lineages I, II, and III, respectively, that were significantly associated with isolation sources (i.e., soil and produce processing facilities), including 450, 50, 29, and 80 over-represented among soil isolates and 452, 0, 7, and 115 over-represented among isolates from produce processing facilities, respectively (Fisher’s exact adjusted *P* < 0.05; Fig. 2a, Fig. S4, Table S8). For *L. seeligeri*, *L. innocua*, and *L. welshimeri*, a total of 6, 357, and 306 accessory genes were significantly associated with sources (i.e., soil, agricultural water, produce processing facilities), respectively (adjusted *P* < 0.05; Fig. 2b, Table S9).

Functional enrichment analysis showed that among source-associated accessory genes, (i) “cell wall/membrane/envelope biogenesis (M)” was significantly enriched in *L. monocytogenes*, *L. monocytogenes* lineage II, *L. innocua*, and *L. welshimeri*, (ii) “Carbohydrate transport and metabolism (G)” was significantly enriched in *L. monocytogenes* lineage III and *L. welshimeri*; and (iii) “Intracellular trafficking, secretion, and vesicular transport (U)” was significantly enriched in *L. welshimeri* (*P* < 0.05; Fig. 2c). Among the 88 source-associated accessory genes annotated as “cell wall/membrane/envelope biogenesis (M)” in *L. monocytogenes*, 12 genes were annotated as a leucine-rich repeat (LRR) protein, all of which were significantly over-represented from soil isolates except for two genes (Table S8). Among the 24 and 15 source-associated accessory genes annotated as “Carbohydrate transport and metabolism (G)” in *L. welshimeri* and *L. monocytogenes* lineage III, at least 6 and 4 genes were involved in the phosphotransferase system (PTS), respectively, nearly all of which were significantly over-represented in food-associated environments (Tables S8, S9). Among the nine source-associated accessory genes annotated as “Intracellular trafficking, secretion, and vesicular transport (U)” in *L. welshimeri*, three genes were annotated as a Type IV secretion system protein (Table S9). These results indicate a strong association between accessory genomes of *Listeria* species, particularly genes involved in cell envelope synthesis and carbohydrate metabolism, and environments.

### Virulence genes, plasmids, and stress survival islets are associated with isolation sources

Many genetic elements (e.g., virulence genes, plasmids, and stress response genes) have been reported to play a role in bacterial adaptation to a particular environment [17, 18]. We thus profiled virulence genes in *L. monocytogenes* and plasmids and stress survival islets in all species studied here. All *L. monocytogenes* isolates from both natural and food-associated environments harbored a putative functional LIPI-1, except for 6 out of 87 lineage I isolates and 5 out of 159 lineage III isolates for which premature stop codon was detected in *actA* gene (Fig. 1a). Internalin genes (*inlABCEFGHJKIP*) were present in most *L. monocytogenes* isolates, with some internalin genes (e.g., *inlF* and *inlG*) showing a lower prevalence particularly among lineage III isolates (Fig. 1a). LIPI-3 was common among lineage I isolates, with a prevalence of 78.2% compared to 6.5% and 3.8% among lineage II and III isolates (Fig. 1a). LIPI-4 was more prevalent in comparison to LIPI-3 and was found in 43.7%, 9.3%, and 43.4% of lineage I, II, and III isolates, respectively (Fig. 1a). All putative functional LIPI-3 and LIPI-4 genes, as well as most putative functional internalin genes (*inlA*, *inlC*, *inlE*, *inlF*, *inlG*, *inlH*, *inlI*, *inlP*), were significantly associated with sources (Fisher’s exact adjusted *P* < 0.05; Fig. S6). Putative functional *inlA* and LIPI-4 genes were significantly over-represented among soil isolates, while putative functional *inlC*, *inlE*, *inlF*, *inlG*, *inlH*, *inlI*, *inlP*, and LIPI-3 genes were significantly over-represented among isolates from produce processing facilities (adjusted *P* < 0.05; Fig. S6). While *inlA* and *inlB* are on the same operon, only *inlA* was overrepresented in soil isolates due to premature codon being present in more isolates from food processing facilities compared to *inlB*.

**Fig. 3:**
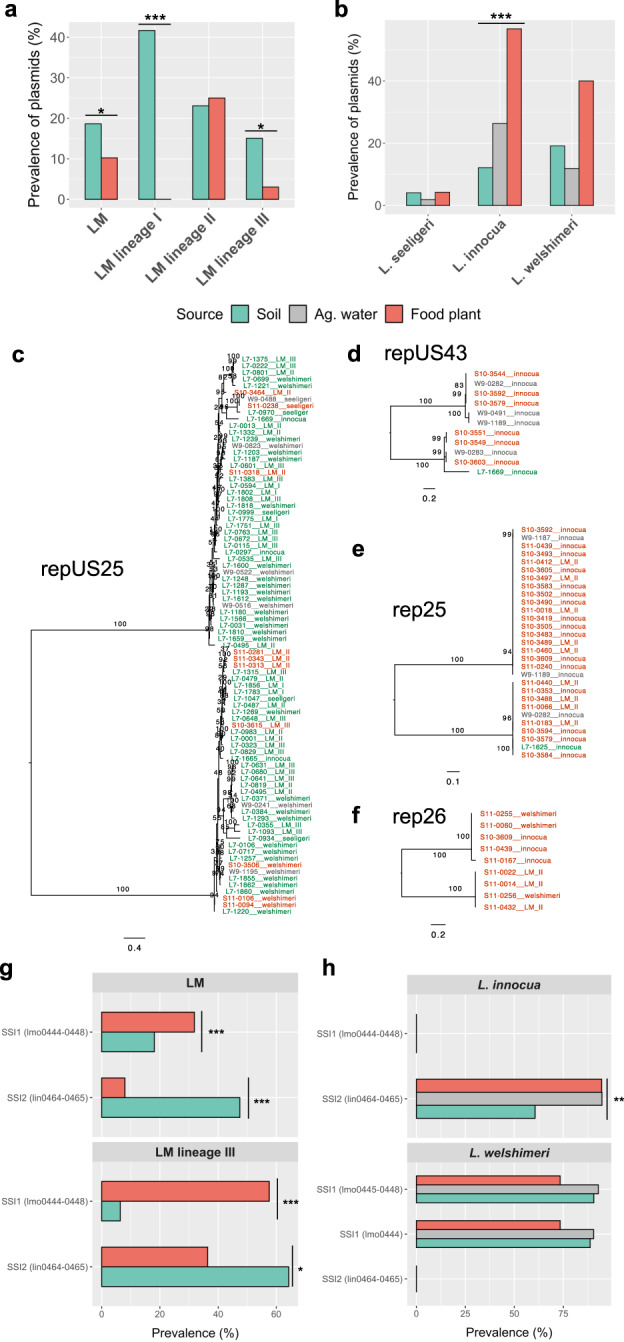
Source association for *Listeria* plasmids and stress survival islets (SSI). Prevalence of (**a**) plasmids among *L. monocytogenes* (LM) and LM lineage I, II, III isolates, (**b**) plasmids among *L. seeligeri*, *L. innocua*, and *L. welshimeri* isolates. Maximum likelihood tree for plasmid group (**c**) repUS25, (**d**) repUS43, (**e**) rep25, (**f**) rep26 based on nucleotide sequences of 84, 11, 29, and 9 *Listeria* isolates repectively with 500 bootstrap repetitions. Bootstrap values are shown on the bifurcation nodes. The tree was rooted by the midpoint. The tips are color coded by isolation sources (green: soil, red: food-processing facilities, and gray: agricultural water). **g** SSI genes among LM and LM lineage III, and (**h**) SSI genes among *L. innocua* and *L. welshimeri* isolates from different sources, including soil, agricultural (ag.) water, and produce processing facilities (food plant). “***”, “**”, and “*” indicates that the distribution of plasmids or SSI genes is significantly dependent on sources at *P* level of 0.001, 0.01, and 0.05, respectively, in Fisher’s exact tests.

Plasmids were not common among *Listeria* isolates, with a prevalence of 14.4% (51/353), 3.5% (7/199), 33.7% (30/89), 19.2% (38/198) in *L. monocytogenes*, *L. seeligeri*, *L. innocua*, and *L. welshimeri*, respectively (Table S10). The frequency of plasmids in *L. monocytogenes*, *L. monocytogenes* lineage I, lineage III, and *L. innocua* was significantly associated with isolation sources (Fisher’s exact *P* < 0.05; Fig. 3a, b). Particularly, plasmids were over-represented among soil isolates of *L. monocytogenes* and lineage III and exclusively present among soil isolates of *L. monocytogenes* lineage I, while for *L. innocua*, plasmids were significantly over-represented among isolates from produce processing facilities (Fig. 3a, b). Plasmids classified into the Inc18 family were highly correlated with a number of source-associated accessory genes in *L. monocytogenes* (10 genes), *L. monocytogenes* lineage I (50 genes), and *L. innocua* (59 genes) (Phi correlation *r* > 0.5; Fig. S8, Table S11). Many of these plasmid-correlated genes were annotated with functions involved in replication, such as resolvase and recombinase, and a few were involved in metal resistance (e.g., arsenic resistance operon repressor) (Table S11). Of note, a total of nine plasmid groups were detected, including rep13, rep25, rep26, rep32, rep33, rep35, rep7a, repUS25, and repUS43. To infer potential horizontal transfer of plasmids across environments and across species, we constructed a gene tree for each of the four plasmid groups that harbored by more than three genomes (rep25, rep26, repUS25, and repUS43). We found that the largest plasmid group, repUS25, was predominately present in soil isolates (81% out of 84 isolates) and exhibited two major clades with a mixture of isolates from both soil and food-associated environments and all four species, *L. monocytogenes*, *L. seeligeri*, *L. welshimeri*, and *L. innocua* (Fig. 3c). The plasmid group repUS43 was predominately present in isolates from food-associated environments (91% out of 11 isolates) and was exclusively detected in *L. innocua* (Fig. 3d). The plasmid group rep25 was also predominately present in isolates from food-associated environment (97% out of 29 isolates) and exhibit two major clades with a mixture of *L. innocua* and *L. monocytogenes* lineage II isolates (Fig. 3e). The plasmid group rep26 was exclusively found in isolates from food processing facilities and formed two major clades, one with *L. welshimeri* and *L. inncoua* isolates and the other with *L. monocytogenes* lineage II and *L. welshimeri* (Fig. 3f). These results suggest that plasmid groups are strongly associated with isolation sources and some plasmids (e.g., repUS25, rep25) may transfer across environments and species in *Listeria*.

In addition, we observed a strong association between putative functional stress survival islets and isolation sources in some *Listeria* taxa. SSI-1 was detected in 24.9% of *L. monocytogenes* isolates including lineages I, II, and III (Fig. S5, Fig. 3g). SSI-1 for *L. monocytogenes* and its lineage III was significantly enriched among isolates from food processing facilities (Fisher’s exact adjusted *P* < 0.05, Fig. 3g). SSI-1 was found in 89.1% of *L. welshimeri* isolates but was not significantly associated with sources (Fig. 3h). The difference in prevalence between SSI-1 lmo0444 and other SSI-1 genes was caused by a premature stop codon present in lmo0444 of one soil isolate and one isolate from agricultural water (Fig. 3h). SSI-2 was detected in 50.1% of *L. monocytogenes* isolates including lineages II and III (Fig. S5, Fig. 3g). SSI-2 for *L. monocytogenes* and its lineage III was significantly enriched among soil isolates (adjusted *P* < 0.05, Fig. 3g). SSI-2, found in 82.0% of *L. innocua* isolates, was significantly over-represented among isolates from agricultural water and produce processing facilities (adjusted *P* < 0.05, Fig. 3h). No putative functional SSI-1 and SSI-2 genes were detected in any *L. innocua* and *L. welshimeri* isolates, respectively, and neither putative functional SSI-1 genes nor SSI-2 genes were detected in any *L. seeligeri* isolates.

### Genetic similarity of soil-dwelling *Listeria* species is correlated with soil properties, climatic factors, and surrounding land use

**Fig. 4:**
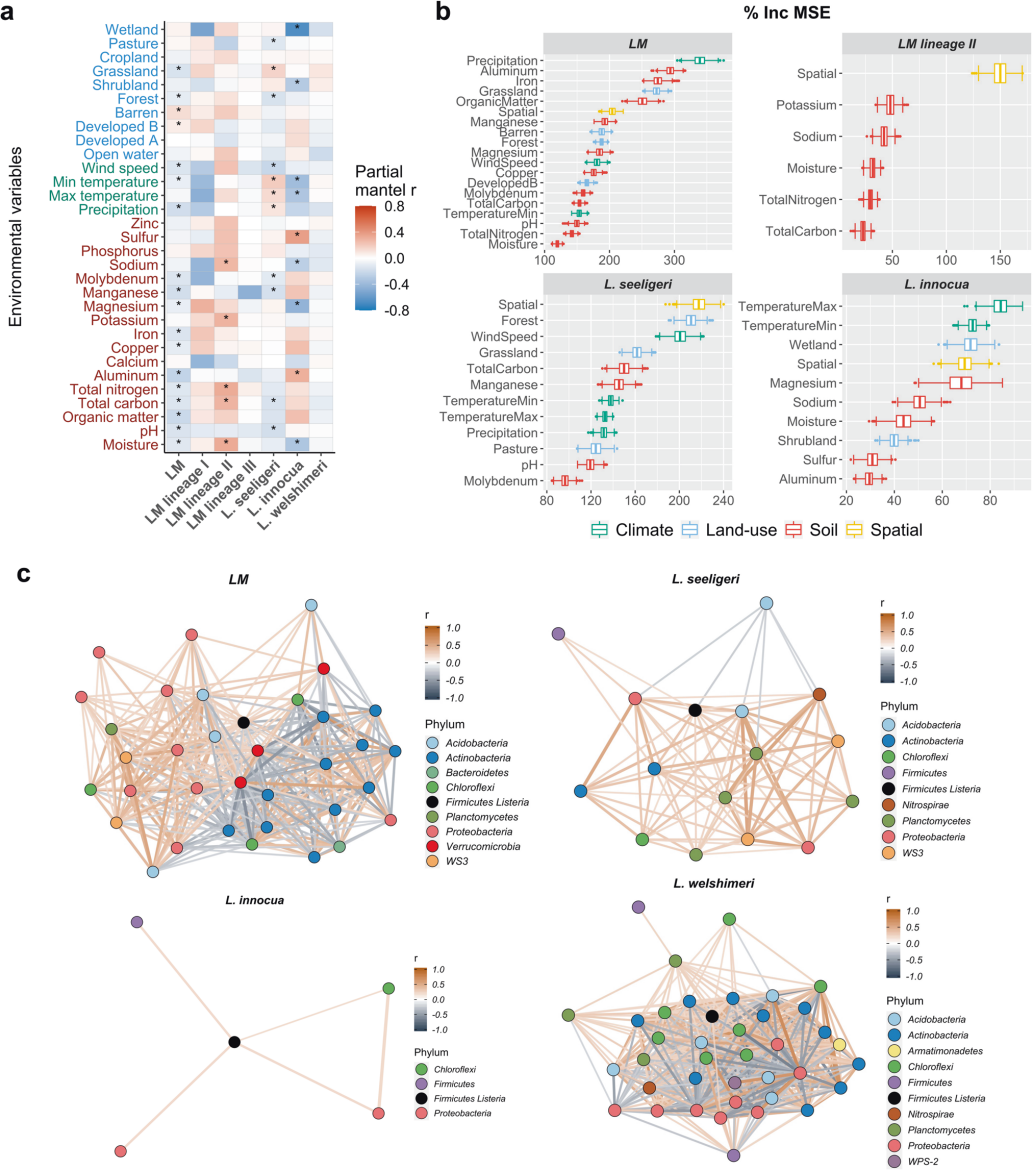
Abiotic and biotic factors that may contribute to the genomic diversification of *Listeria* species. **a** Partial Mantel correlation between ANI of isolates for *L. monocytogenes* (LM), LM lineage I, II, and III, *L. seeligeri*, *L. innocua*, and *L. welshimeri* and geographic distance-correlated dissimilarity of environmental variables. r is the Partial Mantel coefficient. Significant correlations (adjusted *P* < 0.05) are denoted by “*”. Labels for soil, climate, and land-use variables on the *y*-axis are color-coded in red, green, and blue, respectively. Developed A: developed open space with <20% impervious cover; Developed B: developed open space with >20% impervious cover. **b** Variable importance in predicting the ANI of isolates for LM, LM lineage II, *L. seeligeri*, and *L. innocua* based on % Inc MSE index in a random forest model. Abiotic variables on the *y*-axis are sorted in ascending order based on the median % Inc MSE value of 1000 repetitions. “spatial” indicates geographic distance. Minimum and maximum values are depicted by short vertical lines of whiskers; the box signifies the upper and lower quartiles, and the short line within the box signifies the median. Points above and below the whiskers indicate outliers. Boxes and whiskers are color-coded by ecological variable groups. **c** Network of co-occurring bacterial species and LM, *L. seeligeri*, *L. innocua*, and *L. welshimeri*. Each node stands for a bacterial species that had a Phi correlation coefficient (r) > 0.2 or < −0.2 with one *Listeria* species. Nodes representing *Listeria* species are in black (these data are based on culture data generated, not 16 S amplicon sequencing data), and other nodes representing co-occurring bacterial species are color-coded by phylum. An edge stands for the Phi correlation with an *r* > 0.2 or < −0.2 between the two nodes. The thickness of the edge is in proportion to the absolute value of the Phi correlation r. An orange edge represents a positive correlation, while a gray edge represents a negative correlation.

Given the large genomic divergence associated with environments in *Listeria*, we further sought to identify abiotic factors potentially contributing to the diversification. We analyzed the genomic dataset of soil isolates which is coupled with a comprehensive dataset of environmental factors, including geographic location, soil property, climate, and land use [22]. Using partial Mantel tests, we identified several geographical distance-corrected environmental variables that were significantly correlated with genetic similarity of *L. monocytogenes*, *L. monocytogenes* lineage II, *L. seeligeri*, and *L. innocua* based on ANI (adjusted *P* < 0.05) (Fig. 4a, Table S12); these variables were referred to as “abiotic drivers” of the genetic variation in these taxa. The abiotic drivers of *L. monocytogenes* were 11 soil variables (e.g., aluminum, organic matter, manganese), three climatic variables (e.g., precipitation), and four land-use variables (e.g., grassland). All abiotic drivers of *L. monocytogenes* lineage II were soil variables, including total nitrogen, moisture, total carbon, potassium, and sodium. Abiotic drivers of *L. seeligeri* were four soil variables (e.g., pH, manganese), all four climate variables, and three land-use variables (e.g., grassland, pasture). Abiotic drivers of *L. innocua* included five soil variables (e.g., sulfur, magnesium), two climate variables (maximum and minimum temperatures), and two land-use variables (wetland and shrubland). No abiotic drivers were identified for *L. monocytogenes* lineage I and III or *L. welshimeri*.

To quantify the relative importance of the abiotic drivers for *L. monocytogenes*, *L. monocytogenes* lineage II, *L. seeligeri*, and *L. innocua*, we developed random forest models to predict the ANI of isolates representing these taxa using significant environmental variables. Based on both % Inc MSE (Fig. 4b) and Inc Node Purity (Fig. S7), the two most important variables were (i) precipitation and aluminum (for *L. monocytogenes*); (ii) potassium and sodium (for *L. monocytogenes* lineage II); (iii) proximity to forest and wind speed (for *L. seeligeri*), and (iv) annual maximum/minimum temperature and proximity to wetland (for *L. innocua*). These results suggest that genomic variation within *Listeria* species is jointly influenced by geographic location, soil properties, climate, and land use, and the key abiotic factors vary across different taxa.

### *Listeria* shows co-occurrence patterns with other bacterial species in the soil

Besides abiotic factors, biotic factors may also act as selective pressures and contribute to bacterial genomic diversification. We thus further characterized bacterial communities of soil samples selected in this study using 16S rRNA gene amplicon sequencing. The richness and Shannon-Wiener diversity of bacterial OTUs for samples positive for all taxa studied here were significantly higher than those of samples negative for *Listeria* (*t*-test *P* < 0.05 for all; Fig. S9). To identify candidate bacterial species that may contribute to genomic diversification in *Listeria* species, we analyzed the co-occurrence pattern of *Listeria* species and 1,172 bacterial species detected using Phi correlation analysis. A total of 22, 14, 4, and 30 species exhibited a relatively strong positive correlation with *L. monocytogenes*, *L. seeligeri*, *L. innocua*, and *L. welshimeri*, respectively (Phi correlation r > 0.2; Table S13). A large proportion of the species positively correlated with *L. monocytogenes* and *L. innocua* (41% and 50%, respectively) was classified into the phylum *Proteobacteria*, including the families *Hyphomicrobiaceae* and *Rickettsiaceae*; 29% of the species positively correlated with *L. seeligeri* were classified into the phylum *Planctomycetes*, including the family *Pirellulaceae*; and 33% of the species positively correlated with *L. welshimeri* were classified into the phylum *Actinobacteria*, including the family *Pseudonocardiaceae* (Fig. 4c, Table S13). These positively correlated species may occupy similar habitats as these *Listeria* species.

By contrast, a smaller number of species exhibited a relatively strong negative correlation with these four *Listeria* species (12, 1, 0, and 8 species, respectively; Phi correlation *r* < −0.2; Table S13). A total of 83% of the species negatively correlated with *L. monocytogenes* were classified into the phylum *Actinobacteria*, including the family *Geodermatophilaceae* (e.g., *Geodermatophilus obscurus*) and *Pseudonocardiaceae* (e.g., *Pseudonocardia halophobica*); 100% of the species negatively correlated with *L. seeligeri* were classified into the phylum *Acidobacteria*; and 50% the species negatively correlated with *L. welshimeri* was classified into the phylum *Proteobacteria*, including the family *Methylocystaceae* (Fig. 4c, Table S13). For *L. monocytogenes* lineage I, II, and III, a total of 9, 3, and 16 species and a total of 0, 1, and 4 species exhibited a relatively strong positive and negative correlation, respectively (*r* > 0.2 and *r* < −0.2, respectively; Fig. S10, Table S13). These negatively correlated bacterial species may prefer different or distinct habitats than these *Listeria* taxa. In summary, we propose that certain *Proteobacteria* and *Actinobacteria* species are taxa of interest that might pose selective pressures on *Listeria* and contribute to its genome evolution in the soil environment.

## Discussion

Here, we employed in-depth comparative genomic analyses on an extensive genomic dataset of *Listeria* isolates collected by us from soil, agricultural water, and food processing facilities across the US to study genomic diversification of four important *Listeria* species in natural and food-associated environments. Overall, our results suggest that *Listeria* species remodel both core genomes and accessory genomes and differentially adapt to natural and food-associated environments. We hypothesize that such differential adaption exists in *Listeria* from other natural environments (e.g., river water) and food-associated environments as well, driven by different intrinsic and extrinsic factors. Also, our results suggest that the likelihood of the transmission of *Listeria* from natural environments to food-associated environments may be low due to constraints in genetic composition and environmental pressures.

### *Listeria* appears to adapt to natural and food-associated environments through diversifying core genomes at a nucleotide level and gain/loss of accessory genes, particularly those involved in cell envelope synthesis and carbohydrate metabolism

The observed strong associations between genome diversification and isolation sources of *Listeria* species suggest that the splitting of genome diversification between isolates from soil and food-associated environments might be associated with the introduction of *Listeria* from its primary habitat in nature to the food production chain, which may involve lifestyle changes (e.g., from a saprophytic to a facultatively intracellular and pathogenic lifestyle in pathogenetic species [35]). To infer a point in time for this splitting event by including genomes of isolates sampled from a broader range of time points is necessary for future studies.

Both environment-associated core genes and accessory genes identified in this study were enriched for functions involved in the biogenesis of the cell envelope. The cell envelope is the outermost layer that mediates the interaction between microbes and their environments and may, thus, potentially undergo strong selection [36]. Our results are consistent with previous findings that the up-regulation of genes involved in cell envelope stress response in *Listeria* is linked to exposure to food-associated stress conditions [37–40]. For example, in response to benzethonium chloride, which is a quaternary ammonium compound commonly used as a disinfectant in the food processing environment, *L. monocytogenes* was reported to up-regulate several genes and gene pathways directly or indirectly involved in cell wall biosynthesis (e.g., *mnaA* and *tagGH*, which encode proteins involved in teichoic acid biogenesis) [38]. While most of these studies identified the stress response of *Listeria* at the transcriptional level (e.g., differential expression of genes), our results suggest that the gain and loss of genes involved in the cell wall and membrane biogenesis may also be an important mechanism underlining the adaptation of *Listeria* to possible stresses associated with these habitats. A notable group of genes classified as being involved in cell envelope biosynthesis was genes encoding LRR proteins, which are vital to a variety of protein-protein interactions in many bacteria [41, 42]. *L. monocytogenes* encodes a multigene family of LRR-containing LPXTG proteins, including internalins which are used in the adhesion and invasion of host cells [43]. Since internalins tend to be less important to the survival of *L. monocytogenes* in the non-host environment, theoretically, it may be a metabolic cost to maintain these genes in these environments [19]. The underrepresentation of genes putatively encoding LRR proteins in isolates from food processing facilities and the underrepresentation of internalin genes, including *inlC*, *inlE*, *inlF*, *inlG*, *inlH*, *inlI*, *inlP*, in isolates from the soil suggests a higher metabolic cost and loss rate of these genes in *L. monocytogenes* in food processing facilities and soil, respectively, compared to the other environment.

Another interesting function category enriched among environment-associated accessory genes was carbohydrate transport and metabolism. Living a saprotrophic lifestyle, *Listeria* is genetically equipped to access varying carbon sources from multiple non-host environments [44]. The association of carbohydrate transport and metabolism functions with isolation sources may be caused by the differences in abundance and type of carbohydrates found in natural and food-associated environments. In addition, the accessibility of carbohydrates may differ between these two environments; for example, carbohydrates in soil are normally mixed with other organic substances (e.g., residues of plant and microbial materials, humus) [45]. Notably, many environment-associated genes involved in carbohydrate transport and metabolism, including LIPI-4 [46], are PTS genes, which encode functions that mediate the uptake of a variety of sugars [47]. Previous studies have reported higher transcript levels of known activators of multiple PTS systems (e.g., glucose/glucoside, mannose-fructose-sorbose) in soil [48]. Our data suggest that the gain and loss of genes involved in the PTS system likely contribute to the adaptation of *Listeria* to different environments as well.

### Plasmids and stress survival islets may facilitate differential adaptation in *Listeria*

As important drivers of bacterial evolution, plasmids encode a variety of functions allowing for adaption to different stresses in bacteria, including *Listeria* [18, 49]. We found an over-representation of plasmids in *L. monocytogenes*, particularly lineage I and III, from the soil in comparison to those isolated from produce processing facilities. Interestingly, several studies showed that plasmids were over-represented in *L. monocytogenes* isolates from food-associated environments compared with those from clinical cases [50, 51]. It appears that switching habitats from the natural environment to a food-associated environment, then to hosts where they live a facultative pathogenic lifestyle, is accompanied by reduced persistence of plasmids in this pathogen. The maintenance of plasmids is known to be hindered by two major factors: the burden generated by plasmids in the host cell and the loss rate of plasmids during cell division [49]. Our results suggest that the metabolic burden and/or the loss rate of plasmids may be higher for *L. monocytogenes* in a food-associated environment, possibly caused by unfavorable conditions compared to the natural environment. However, we observed an opposite pattern in *L. innocua*, i.e., an over-representation of plasmids in isolates from food-associated environments compared to the natural environment. Interestingly, we detected a high correlation between plasmid family Inc18 and several environment-associated genes encoding functions related to metal resistance (e.g., copper and arsenic resistance) in this species, consistent with previous detection of cadmium, copper, and arsenite resistance genes on *Listeria* plasmids [52]. These results suggest that maintaining a high abundance of plasmids in a food-associated environment may increase the fitness of *L. innocua*, putatively in response to heavy metal stresses.

Stress survival islets encode functions that are expected to facilitate *Listeria* growth in suboptimal conditions [53, 54]. So far, two stress survival islets have been identified in *Listeria*; SSI-1, a five‐gene islet (lmo0444 - lmo0448), was reported to facilitate *Listeria* growth at low pH and high salt concentrations [53], and SSI-2, a two-gene islet (lin0464 - lin0465), was reported to be beneficial at high pH and in oxidative conditions [54]. Previous studies suggested that SSI-1 and SSI-2 genes are associated with specific subgroups of *L. monocytogenes* and *Listeria* species [54–56]. For instance, for *Listeria* isolated from produce processing environments, SSI-1 was especially prevalent among *L. monocytogenes* sequence type (ST) 14 isolates, while SSI-2 was found predominately in *L. monocytogenes* ST121 and *L. innocua* isolates [55]. Here, we show that SSI-1 and SSI-2 are also associated with environments. The over-representation of SSI-1 in *L. monocytogenes* lineage III isolates from food-associated environments possibly allows it to cope with acid and osmotic stresses present in these environments, while the over-representation of SSI-2 in *L. innocua* isolates from food-associated environments possibly allows it to cope with alkaline and oxidative stresses present in these environments.

### Different combinations of soil property, climate, and land-use factors and *Proteobacteria* and *Actinobacteria* species may act on different *Listeria* species in the soil as stressors

The two environments studied here (i.e., natural environment [soil] and food-associated environments [agricultural water and produce processing facilities]) represent distinct conditions for the survival and fate of *Listeria*. While soil is a heterogeneous environment and unexpected environmental changes may occur, its physicochemical properties, in general, remain stable [20, 21]. By comparison, agricultural water and produce processing facilities may represent a more stressful and less stable environment (for example, due to large fluctuations in temperature in agricultural water and large fluctuations in pH and nutrient availability, and the presence of chemical and antimicrobial compounds in produce processing facilities) [16]. Our data show that the genomic divergence of *Listeria* species and *L. monocytogenes* lineages is associated with different combinations of soil, climate, and land-use variables. Besides some edaphic factors (e.g., pH, moisture) known to be important to *L. monocytogenes* [11, 57, 58], we identified several other soil variables (e.g., concentrations of aluminum, iron, organic matter) as well as climate and land-use variables (e.g., precipitation, grassland proportion) that haven’t been frequently linked to the ecology and evolution of *L. monocytogenes* in soil. In addition, we found that climate variables were particularly important for non-pathogenic *Listeria* species, including *L. seeligeri* and *L. innocua*, which may directly or indirectly trigger adaptations in these species [59].

*Listeria* in soil interacts not only with abiotic elements but also with other organisms [44]. Such interactions may influence the assembly of *Listeria* populations and contribute to their adaptation to the soil environment, which indirectly impacts the genomic diversity of *Listeria* populations. A number of *Proteobacteria* species were found to be positively correlated with *Listeria* species detection. While, in general, positive associations between two bacterial taxa are hypothesized to be caused by cooperation (e.g., for nutrient acquisition), we were unable to identify any mechanisms that may facilitate direct cooperation between *Listeria* and *Proteobacteria* species. However, *Proteobacteria* produce a variety of polyketides and non-ribosomal peptides, which may inhibit other bacteria [60] that could be competitors of *Listeria*. Interestingly, *Proteobacteria* was also previously found to be the most abundant phylum in bacterial communities of floor drains in a food processing environment contaminated by *L. monocytogenes* [61]. In addition, a large proportion of species negatively correlated with *L. monocytogenes* were classified into *Actinobacteria*. Consistent with our finding, a previous study reported that exopolysaccharide from *Bifidobacterium bifidum*, an *Actinobacteria* species, showed antibacterial activity against several pathogenic bacteria, including *L. monocytogenes* [62]. While our findings shed light on potential interactions of *Listeria* with other bacteria in the soil environment, they are limited to correlations at a species level. Investigations on the causation of microbial communities on the adaptation and genomic diversification of *Listeria* at a finer taxonomic/phylogenetic level (e.g., strains) are needed to better elucidate the mechanisms behind these interactions.

Importantly, these abiotic and biotic factors associated with *Listeria* species may act as external constraints that limit effective and efficient transmission of *Listeria* between natural and food-associated environments, along with source-associated genetic variances acting as intrinsic constraints. While the likelihood that *Listeria* frequently transmits between natural and food-associated environments tends to be low, in *L. monocytogenes* lineage I, which mainly causes clinical infections, we indeed detected 31 isolates from produce facilities that were closely related to one or more soil isolates from NY, MD, IA, and MN. For long-distance *L. monocytogenes* transmission from soil to produce processing facilities, the transmission route that we hypothesize is the soil in the natural environment—adjacent produce fields (e.g., via wild-animal feces deposition)—produce contamination—transportation of contaminated produce to produce processing facilities. It is also possible that *L. monocytogenes* may transmit directly from the soil in the natural environment to nearby produce processing facilities due to human activities (e.g., via the dirt on the shoes). Our results suggest that the incidence of dissemination from natural environments to food-associated environments is not zero, consistent with previous inferences [63].

## Conclusions

Overall, through comparative genomic analysis of *Listeria* isolates from natural and food-associated environments, we detected strong evidence of differential adaptions in both pathogenic and non-pathogenic species of *Listeria*. Intrinsic factors underlying this adaptation included diversification of core genomes and gain and loss of accessory genes, particularly those involved in cell envelope biogenesis and carbohydrate metabolism, as well as plasmids and stress survival islets. With machine learning, these intrinsic genetic variants (e.g., cgMLST) can accurately predict the isolation sources of *Listeria* isolates at a fine taxonomic level, which will benefit the source tracking of future foodborne outbreaks and food contamination caused by *L. monocytogenes*. In addition, we also identified a number of extrinsic factors, including soil, climate, and land-use parameters, and other bacterial species, particularly those representing *Proteobacteria* and *Actinobacteria*, that may contribute to the differential adaptations in *Listeria* species. These intrinsic and extrinsic factors also represent potential novel barriers that limit *Listeria* species, including the foodborne pathogen *L. monocytogenes*, to transmit from natural to food-associated environments in addition to the commonly recognized geographic barriers.

## Supplementary Information

Supplementary information

Large tables

## Data Availability

The datasets used in this study include data from isolates collected from (i) soil in the natural environment across the United States, (ii) agricultural water, and (iii) produce processing facilities. The raw reads and assembled genomes for isolates in sets (i) and (ii) have been deposited in the National Center for Biotechnology Information’s (NCBI) SRA and NCBI DDBJ/ENA/GenBank, respectively, under accession numbers listed in Table S1. Due to the data sensitivity and privacy, raw reads and assembled genomes for isolates in set (iii) were not uploaded to NCBI; however, this data is available on the Cornell University eCommons repository: 10.7298/74sp-fg52. 16 S rRNA sequencing reads were deposited at NCBI Sequence Read Archive under accession number PRJNA749132.
