# Supplementary material for: Comparative genomics unveils extensive genomic variation between populations of Listeria species in natural and food-associated environments
Source: ISME Commun. 2023 Aug 19;3:85. doi: 10.1038/s43705-023-00293-x (PMC10439904; doi:10.1038/s43705-023-00293-x)
Supplement: Supplementary file 1 — Supplementary information [file 43705_2023_293_MOESM1_ESM.pdf]

Supplementary Methods

DNA amplification

Total DNA for each sample was used to amplify the V4 region of the 16S rRNA gene, using the updated 515F and 806R primers (5'- GTGYCAGCMGCCGCGGTAA-3' and 5'-GGACTACNVGGGTWTCTAAT-3', respectively) with the added appropriate 5' Illumina Nextera adapters, as described in Illumina 16S Amplicon Protocol of Earth Microbiome Project (http://www.earthmicrobiome.org/protocols-and-standards/16s/). Successful amplification was confirmed if agarose gel electrophoresis showed a strong band indicative of a DNA fragment of around 350 bp and DNA products passed the quality control ($A_{260/280}$ ~1.80 and $A_{260/230}$ ~2.0) using NanoDrop. For each DNA sample, to avoid the bias caused by a single amplification reaction, two PCR amplification reactions were performed; amplified DNA was pooled, followed by DNA barcoding.

Sequencing read processing

Raw reads were denoised using DADA2, yielding a range of sequencing reads, among samples, from 3,187 to 37,495 (**Table S2**). Based on the alpha rarefaction curve, the Shannon-Wiener diversity saturated at a sequencing depth of near 5,000 reads (**Fig. S1**). Thus, samples were rarefied to 5,000 reads to normalize the sequencing depth. Four samples (two each that tested positive and negative for *Listeria*) that had < 5,000 reads were excluded from analyses (**Table S2**). Sequences with a similarity > 0.97 were clustered *de novo* into operational taxonomic units (OTUs) using q2-vsearch. Taxonomic classification of each OTU was performed by comparing sequences against the pre-trained classifiers Greengenes 13_8 using classify-sklearn. Non-bacterial OTUs and singletons (i.e., OTUs present in one sample with 1 read sequenced) were excluded. OTUs were further collapsed to species-level based on annotation in QIIME2.

Identification of significantly enriched COG functions

To identify clusters of orthologous gene (COG) function categories that were significantly enriched among source-associated accessory genes for each species and each *L. monocytogenes* lineage, a binomial distribution model was used to compare the frequency of each COG function among source-associated orthologous genes with the frequency among all orthologous genes of each *Listeri*a taxon. using the formula below:

$$\mathrm{i} = \frac{n - p * N}{\sqrt{p * (1 - p) * N}}$$

where *n* is the observed count of source-associated genes belonging to each COG functional category, *N* is the total count of source-associated genes, *p* is the frequency of genes belonging to each COG category among all genes, and *i* is the enrichment index, which represents the multiplier of standard deviation in the binomial distribution. A value of > 2 for the enrichment index indicates a significant enrichment at a *P* value of < 0.05.

Supplementary Figures

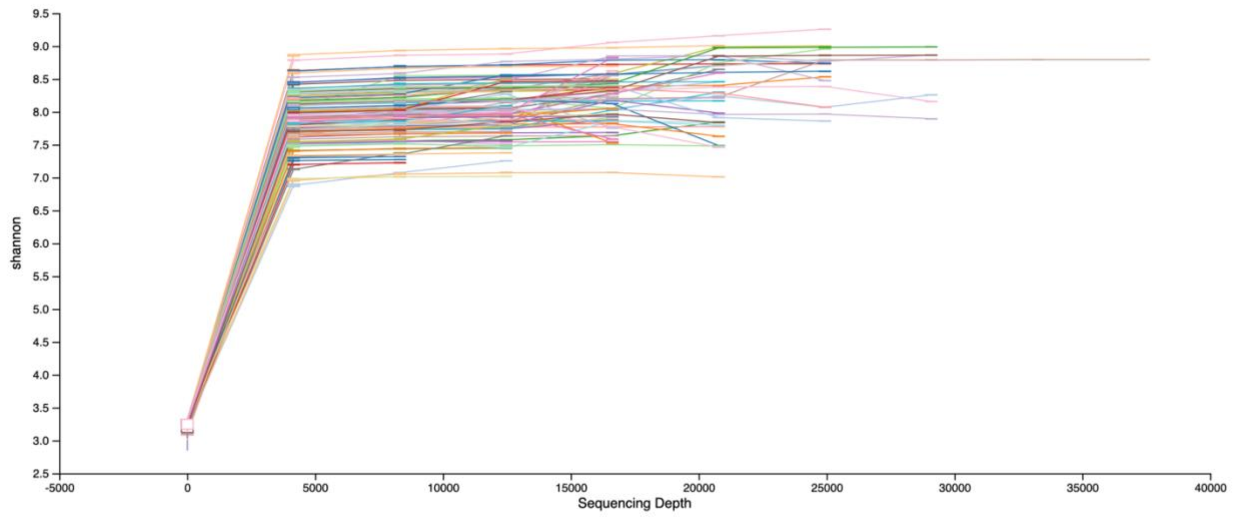

Fig. S1 Alpha rarefaction curve of Shannon-Wiener diversity of OTUs with the increase of sequencing depth for 622 samples. The curves are color-coded by samples. The sequencing depth is denoted by the number of raw reads.

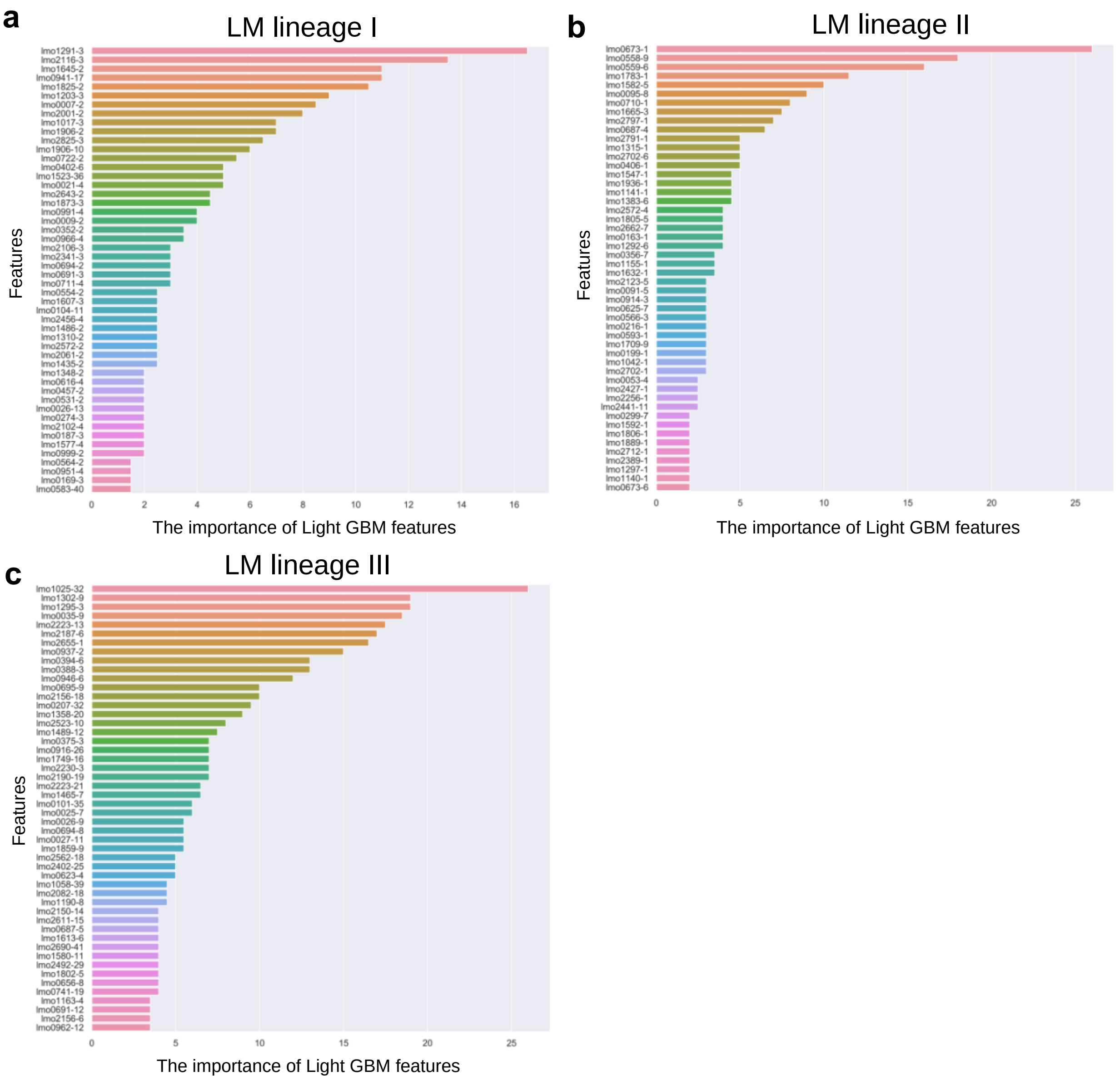

Fig. S2 The top 50 most important features based on averaged importance scores of two folds in the cross validation using LightGBM classifier for source prediction of *L. monocytogenes* (LM) **a** lineage I, **b** lineage II, and **c** lineage III.

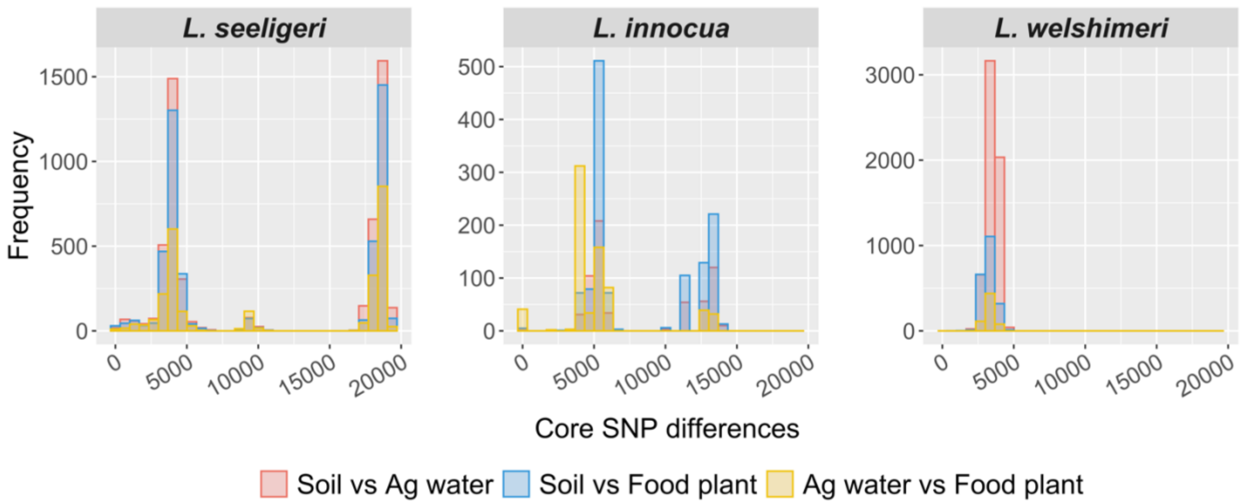

Fig. S3 Frequency of core SNP differences between pairs of isolates from soil and agricultural (ag.) water isolates (red), soil and produce processing facility (food plant) isolates (blue), and ag. water and produce processing facility isolates (yellow) of *L. seeligeri*, *L. innocua*, and *L. welshimeri*.

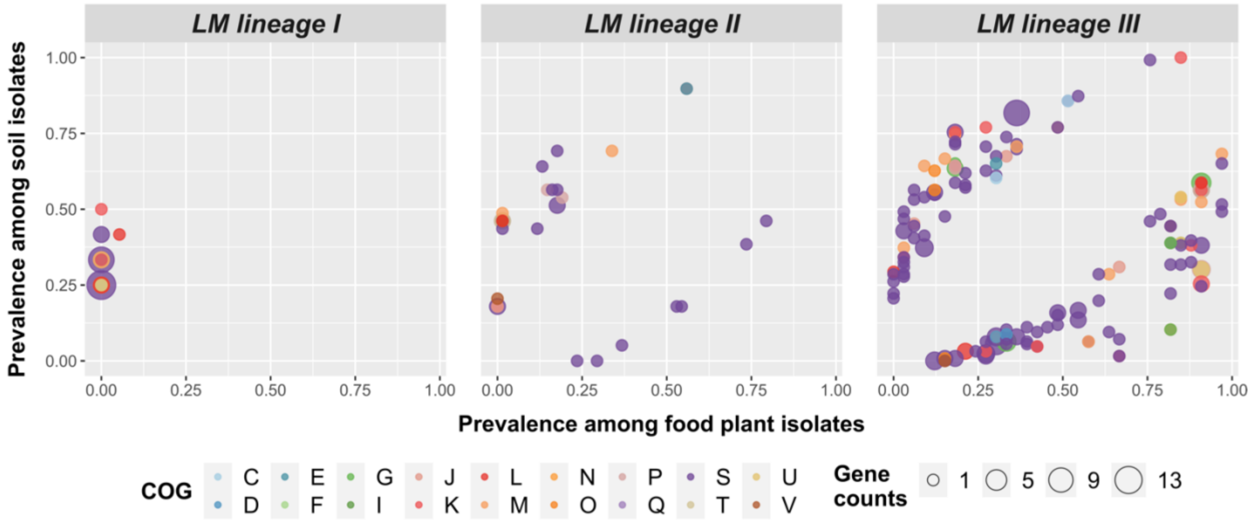

Fig. S4 Prevalence of source-associated orthologous genes between isolates from soil and produce processing facilities (food plant) for *L. monocytogenes* (LM) lineage I, II, and III. Dots are color-coded by COG functional categories. The size of the dots is in proportion to the number of genes annotated as one COG category. Description of the abbreviations of COG categories was detailed in the legend of Fig. 2.

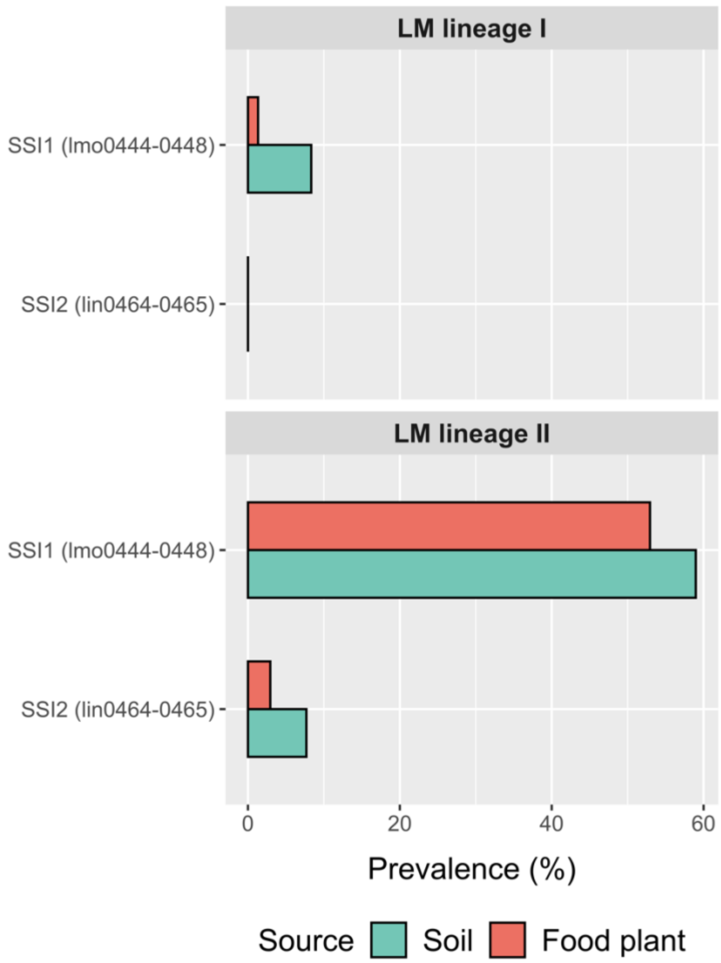

Fig. S5 Prevalence of stress survival islet (SSI) genes among *L. monocytogenes* (LM) lineage I and II isolates from soil and produce processing facilities (food plant).

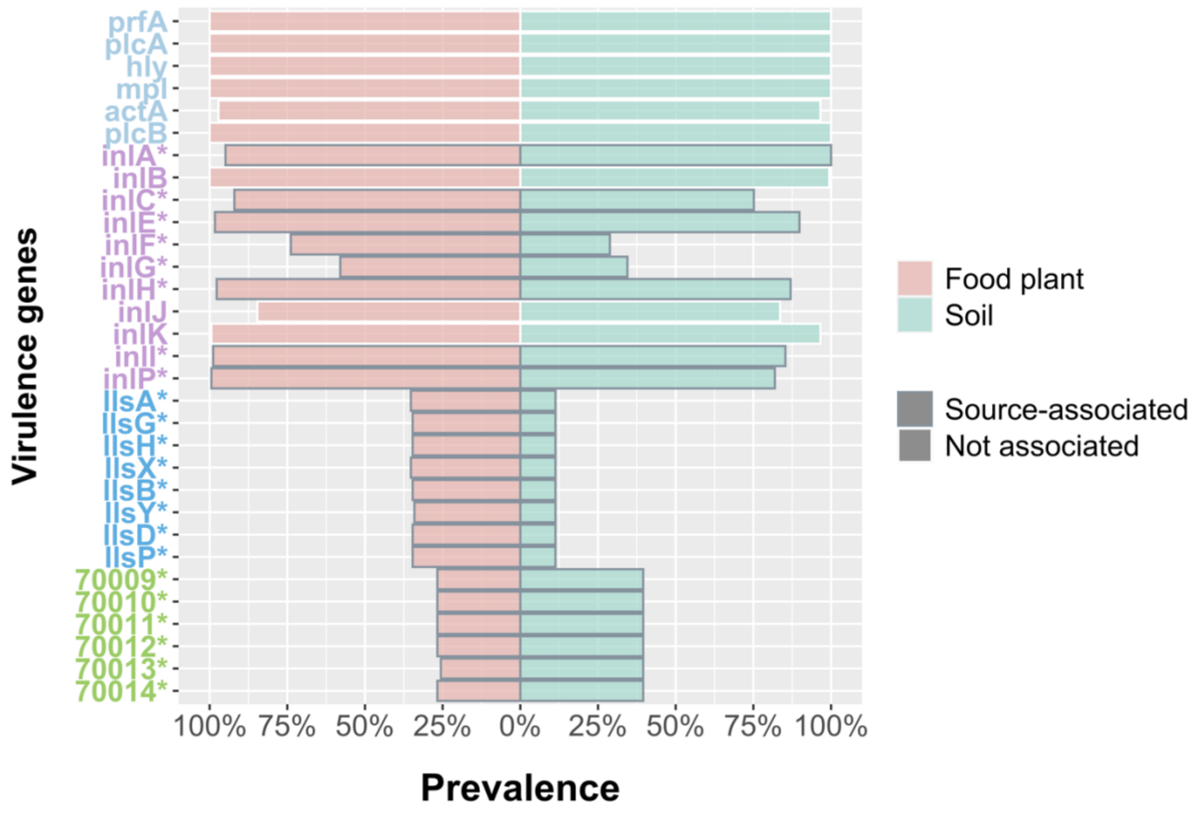

Fig. S6 Prevalence of putative functional virulence genes of LIPI-1, internalin, LIPI-3, and LIPI-4 among *L. monocytogenes* soil and produce processing facility (food plant) isolates. Genes with "*" in the name on the y axis and with grey bar border indicate that the prevalence is significantly associated with the source of isolates based on Fisher's exact tests after BH FDR correction (adjusted $P < 0.05$). Labels of y-axis for virulence genes of LIPI-1, internalin, LIPI-3, and LIPI-4 are in light blue, purple, blue, and green, respectively.

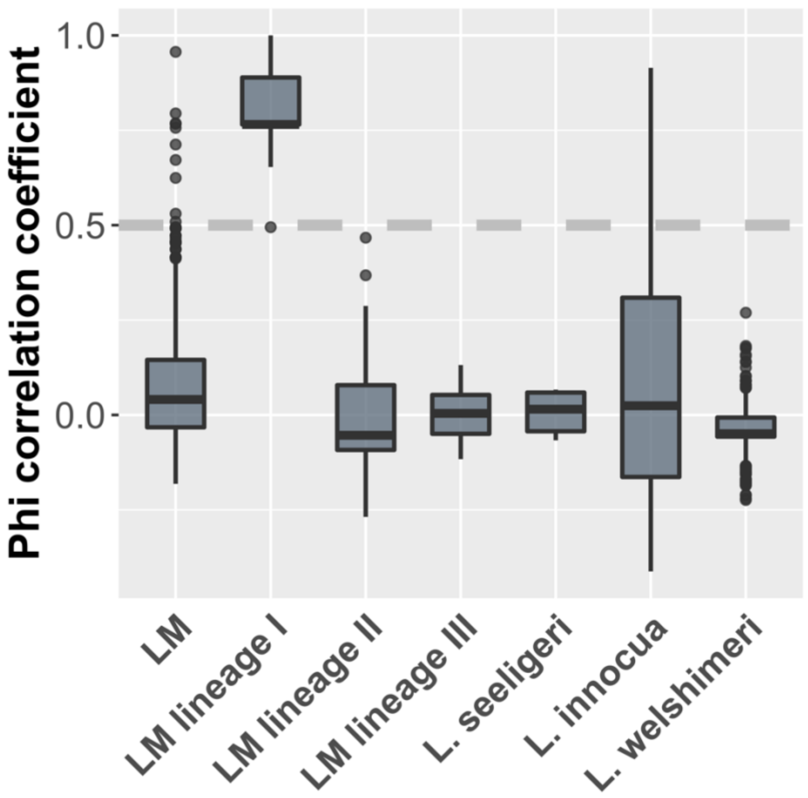

Fig. S7 Distribution of Phi correlation coefficients between source-associated genes and plasmid family Inc18 based on the presence/absence matrix. Minimum and maximum values are depicted by ends of whiskers; the box represents the upper and lower quartiles, and the median is denoted by a short line within the box. Dots above and below the whiskers indicate outliers. The grey dashed line indicates a coefficient of 0.5.

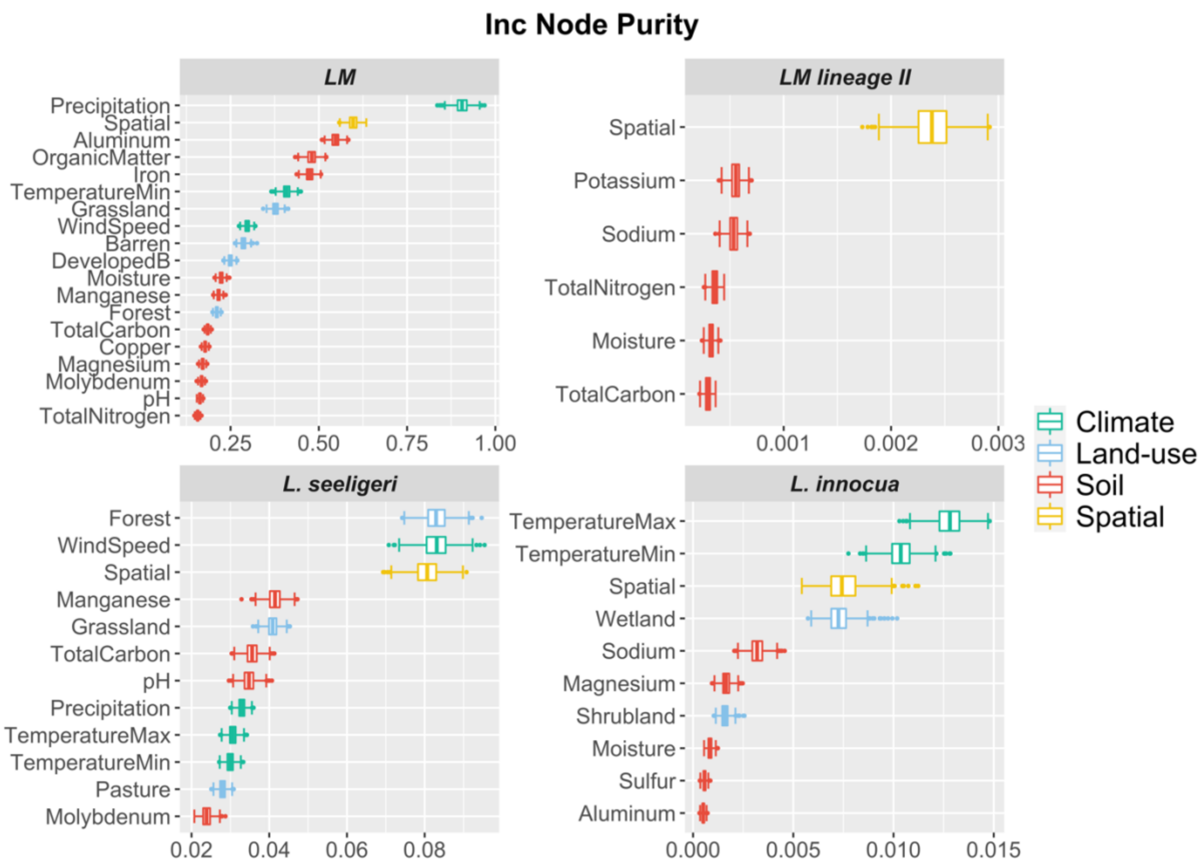

Fig. S8 Variable importance in predicting ANI of isolates for *L. monocytogenes* (LM), LM lineage II, *L. seeligeri*, and *L. innocua* based on Inc Node Purity index in a random forest model. Ecological variables on the y-axis are sorted in ascending order based on the median Inc Node Purity value of 1,000 repetitions. Minimum and maximum values are depicted by short vertical lines of whiskers; the box signifies the upper and lower quartiles, and the short line within the box signifies the median. Dots above and below the whiskers indicate outliers. Boxes and whiskers are color-coded by ecological variable groups. Developed A: developed open space with < 20% impervious cover; Developed B: developed open space with >20% impervious cover.

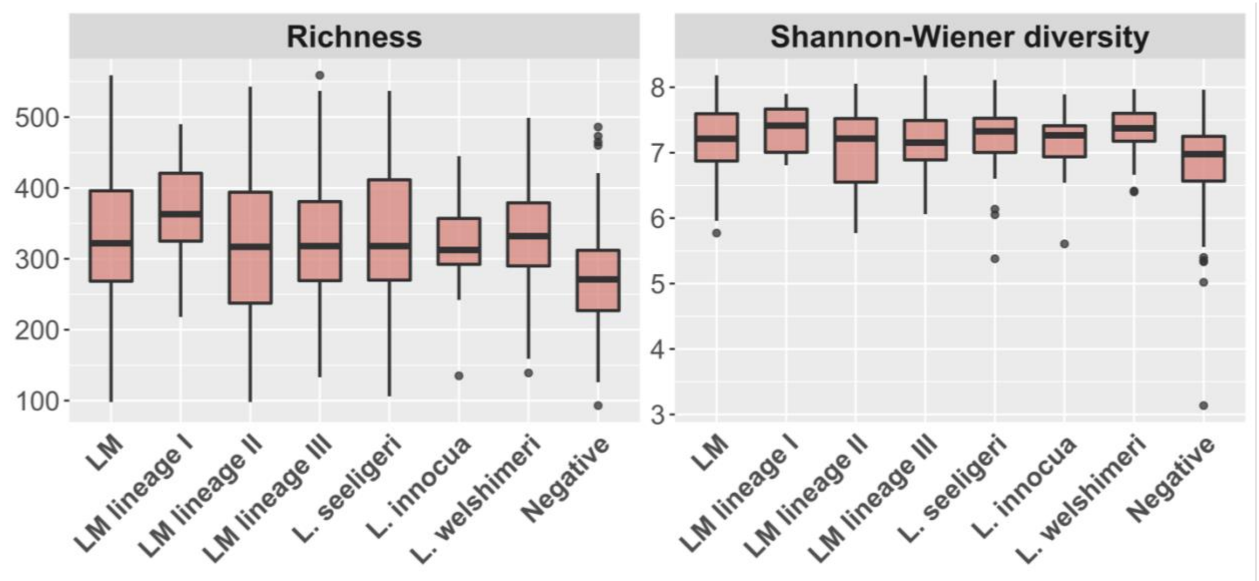

Fig. S9 Distribution of richness and Shannon-Wiener diversity of bacterial OTUs for samples positive for *L. monocytogenes* (*LM*), *LM* lineage I, II, III, *L. seeligeri*, *L. innocua*, and *L. welshimeri*, and for samples negative for *Listeria*. Minimum and maximum values are depicted by ends of whiskers; the box signifies the upper and lower quartiles, and the median is represented by a short line within the box. Dots above and below the whiskers indicate outliers.

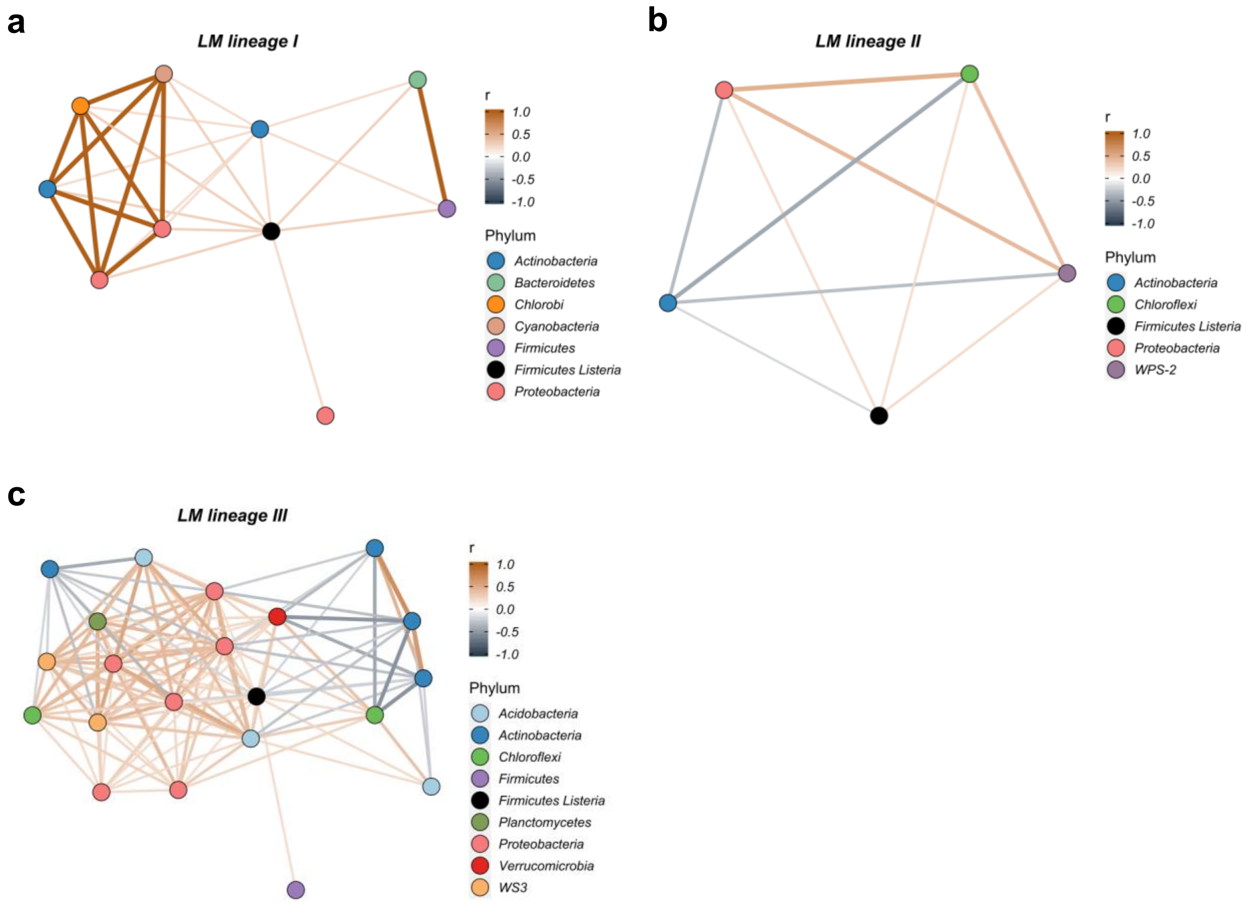

Fig. S10 Network of co-occurring bacterial species and *L. monocytogenes* (*LM*) **a** lineage I, **b** lineage II, and **c** lineage III. Each node stands for a bacterial species which showed a Phi correlation coefficient > 0.2 or < -0.2 with one LM lineage. Nodes representing LM lineages are in black and other nodes representing co-occurring bacterial species are color-coded by phylum. An edge stands for the Phi correlation with a coefficient > 0.2 or < -0.2 between the two nodes. The thickness of the edge is in proportion to the absolute value of the Phi correlation coefficient. An orange edge represents a positive correlation, while a grey edge represents a negative correlation.
